# Long-term survival outcomes of allo-HCT in AML with fludarabine/melphalan conditioning and tacrolimus/sirolimus GVHD prophylaxis

**DOI:** 10.1038/s41409-025-02738-4

**Published:** 2025-11-18

**Authors:** Amandeep Salhotra, Dongyun Yang, Monzr M. Al Malki, Sally Mokhtari, Diana Knobler, Vaibhav Agarwal, Karamjeet Sandhu, Gabriel Park, Ahmed Aribi, Haris Ali, Ibrahim Aldoss, Salman Otoukesh, Shukaib Arslan, Brian Ball, Paul Koller, Idoroenyi Amanam, Hoda Pourhassan, Amanda Blackmon, Pamela Becker, Vinod Pullarkat, Andrew S. Artz, Eileen Smith, Guido Marcucci, Stephen Forman, Anthony Stein, Ryotaro Nakamura

**Affiliations:** 1 Department of Hematology & Hematopoietic Cell Transplantation, City of Hope, Duarte, CA USA; 2 Department of Biostatistics, City of Hope, Duarte, CA USA; 3 Clinical Translational Project Development, City of Hope, Duarte, CA USA; 4 Department of Pharmacy, City of Hope, Duarte, CA USA

**Keywords:** Acute myeloid leukaemia, Chemotherapy

## Abstract

Allogeneic hematopoietic cell transplantation (allo-HCT) is increasingly offered as a consolidation strategy for older/infirm patients with acute myeloid leukemia (AML). Fludarabine/melphalan (Flu/Mel) conditioning is associated with effective disease control but results in significant toxicity and non-relapse mortality (NRM) when combined with calcineurin-inhibitors plus methotrexate or mycophenolate mofetil. Flu/Mel with alternative graft-versus-host disease (GVHD) prophylaxis may be better tolerated and result in superior outcomes in patients with AML. In this single-center retrospective analysis, we analyzed long-term outcomes of patients with AML (n = 342) who underwent allo-HCT with Flu/Mel conditioning and tacrolimus/sirolimus (Tac/Sir)-based GVHD prophylaxis from 2008-2019 at City of Hope. Patient median age was 63 years (range: 23–78), with 37% having high-very high Disease Risk Index (DRI) and 42% with HCT-Comorbidity Index (CI) ≥ 3. Five-year overall survival (OS: primary objective) was 55% (95% CI: 49–61%) among all patients and 70% (95% CI: 55–81%) in patients ≥70 years old. Only presence of active disease correlated with lower 5-year OS on multivariate analysis (HR = 1.95; p < .001). Five-year NRM was 24% (95% CI: 19–29%) among all patients and 21% (95% CI: 11–34%) in those ≥70 years old. In conclusion, Flu/Mel conditioning with Tac/Sir GVHD prophylaxis is associated with favorable OS and acceptable NRM, even in older/infirm patients with AML.

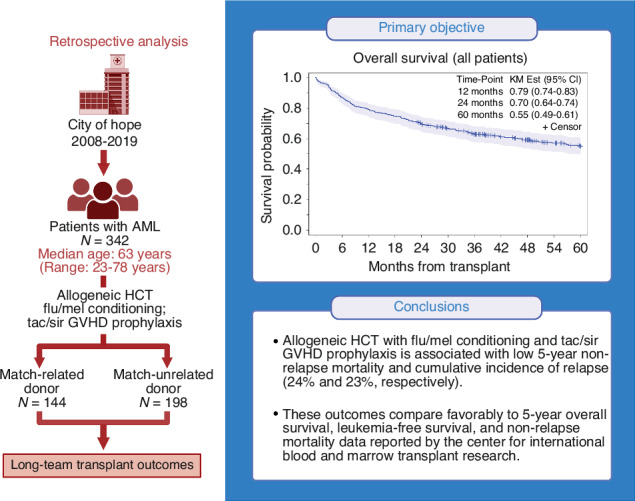

## Introduction

Reduced intensity conditioning (RIC) regimens have expanded access to allogeneic hematopoietic cell transplantation (allo-HCT) for older and infirm patients with hematologic malignancies who are otherwise ineligible for allo-HCT with myeloablative conditioning (MAC) [1]. The most recent data from the Center for International Blood and Marrow Registry (CIBMTR) shows that patients ≥65 years old now comprise one-third of all patients undergoing allo-HCT [2].

Fludarabine (Flu), busulfan (Bu), or melphalan (Mel) are the two commonly used RIC regimens in the United States. Zhou et al. reported outcomes of patients with acute myeloid leukemia (AML; median age: 60 years) who underwent allo-HCT with either Flu/Mel (n = 622) or Flu/Bu (n = 791) and demonstrated similar 5-year overall survival (OS) of 29% and 27% (p = 0.57), respectively [3]. Flu/Mel was associated with lower OS at day +100 post-HCT due to increased non-relapse mortality (NRM), but this was balanced by lower 5-year cumulative incidence of relapse (CIR). Eapen et al. also reported leukemia-free survival (LFS) in patients with AML or myelodysplastic syndrome (MDS; n = 1258, median age: 58 years) who underwent allo-HCT with Flu/Mel (median age: 63 years) or MAC regimens, confirming the efficacy of this regimen in aggressive myeloid malignancies [4]. Finally, Ciurea et al. reported that in AML patients >60 years undergoing allo-HCT with four common RIC regimens and showed superior 5-year progression-free survival in patients conditioned with Flu/Mel [5]. Together, these data suggest that Flu/Mel provides greater long-term disease control but results in higher NRM (up to day +100) compared to Flu/Bu. It is worth noting that the graft-versus-host disease (GVHD) prophylaxis used in these studies was exclusively calcineurin inhibitors (CNI) with methotrexate (MTX) or mycophenolate mofetil (MMF). It is possible that Flu/Mel combined with alternative GVHD prophylaxis may be better tolerated and result in superior outcomes.

Tacrolimus/sirolimus (Tac/Sir)-based GVHD prophylaxis combined with low-dose MTX was first developed in patients undergoing MAC-HCT using bone marrow graft from an HLA-matched unrelated donor (MUD) or single antigen-mismatched unrelated donor (MMUD) [6]. Subsequently, the regimen omitted MTX in matched donors in MAC [7] and RIC-HCT [8, 9]. Four randomized trials evaluated use of Tac/Sir-based prophylaxis in MAC-HCT from matched related donors (MRD) [10], RIC-HCT for lymphoma [11], MAC/RIC-HCT for any hematologic malignancies [12], and in RIC-HCT combined with bortezomib [13]. None of these trials demonstrated improvement in OS using Tac/Siro, but two studies showed improved GVHD rates [11, 12]. Of note, the RIC regimen used in these trials was predominantly Flu/Bu. Khimani et al. evaluated 707 patients (Tac/Sir: n = 293, Tac/MTX: n = 414) who underwent MAC- or RIC-HCT and showed that Tac/Sir GVHD prophylaxis is associated with reduced acute GVHD (aGVHD) and improved OS among patients with HCT-comorbidity index (CI) ≥ 4 [14], with comparable toxicity to Tac/MTX.

At City of Hope, we adopted Tac/Sir-based GVHD prophylaxis with Flu/Mel or total-body irradiation/etoposide conditioning, demonstrating low rates of aGVHD and day +100 NRM in the MRD setting [15]. Subsequently, we expanded use of Flu/Mel with Tac/Sir to MUD-HCT and reported favorable outcomes in patients with MDS [16], myelofibrosis [17], acute lymphoblastic leukemia [18], non-Hodgkin lymphoma [19], and AML with MAC [20].

In this single-institution retrospective study, we conducted a chart analysis of patients with AML who underwent allo-HCT with Flu/Mel conditioning and Tac/Sir GVHD prophylaxis. With a primary endpoint of 5-year OS in the entire cohort and in special subgroups of interest, we aimed to determine long-term clinical outcomes and describe secular trends in patient demographics over time by comparing early (2008–2012) and recent (2013–2019) transplant eras.

## Patients and methods

### Study population

We reviewed medical records of patients with AML (n = 342) who underwent allo-HCT with Flu/Mel conditioning and Tac/Sir GVHD prophylaxis between 2008 and 2019. Both MRD (n = 144) and MUD (n = 198) recipients were included and in accordance with institutional guidelines, a MUD was defined as any unrelated donor who was HLA-matched at high resolution (10/10 match at HLA-A, -B, -C, -DR-1 and -DQ). MUD grafts were obtained from the National Marrow Donor Program. This study was reviewed and approved by the City of Hope Institutional Review Board.

### Transplant procedures

This was conducted per institutional standard. Briefly, Flu was given intravenously (IV) at 25 mg/m²/dose for 5 days, and a single dose of Mel was given IV at 140 mg/m². GVHD prophylaxis consisted of Tac (starting dose of 0.02 mg/kg by continuous IV infusion) and Sir (given at a 12 mg loading dose followed by 4 mg oral daily dose), both starting 2 days before HCT. Subsequent Tac/Sir dosing was based on therapeutic drug monitoring to maintain levels within the recommended therapeutic range (therapeutic trough levels 5–10 ng/ml for both Tac and Sir). Immunosuppression taper after day +100 post-HCT was per the treating physician’s discretion. Ursodiol was given as sinusoidal obstruction syndrome prophylaxis. Infection prophylaxis consisted of acyclovir (400 mg/m^2^) starting on day −1 and micafungin (100 mg) given IV daily. Pneumocystis Jirovecii pneumonia prophylaxis consisted of trimethoprim/sulfamethoxazole starting on the first day of conditioning until day −3 pre-HCT and resuming on day +21 post-HCT. CMV monitoring was started on day +21 post-HCT and continued per SOP.

### Outcome definitions

OS was defined as the time from transplantation to death or censored at the last follow-up in patients known to be alive. Leukemia-free survival (LFS) was calculated from the time of transplantation to the first observation of disease recurrence or death, whichever occurred first. LFS was censored at the last follow-up if the patient remained alive and leukemia-free. GVHD-free, relapse-free survival (GRFS) was defined as the time from transplantation to the earliest occurrence of grade III-IV aGVHD, moderate or severe chronic GVHD (cGVHD), disease relapse, or death. GRFS was censored if patients were alive and free of events of interest. Relapse/progression was defined as the time from transplantation to relapse or progression, with NRM as a competing risk. NRM was defined as the time from transplantation to death from any cause other than relapse/progression, with relapse or progression as a competing risk. aGVHD was defined as the time to onset of grade II-IV aGVHD, with relapse and death as competing events.

### Statistical analysis

Descriptive statistics were used to summarize baseline patient demographics, treatment, and disease characteristics. Kaplan–Meier curves and the log-rank test were used to evaluate OS, LFS, and GRFS. Cumulative incidence curves and the Gray's test were used to examine the differences in relapse rates and NRM. The assumptions of proportionality for Cox regression and Fine and Gray models were checked by corresponding tests and plots of the scaled Schoenfeld residuals or the cumulative sums of residuals whenever appropriate [21]. Dongyun Yang, Ph.D., analyzed the data. The authors had access to the primary data.

## Results

### Patient and transplant characteristics

Characteristics for all patients are summarized in Table 1. Briefly, the median age at allo-HCT was 63 years (range: 23–78). Eleven patients were ≤40 years old and received Flu/Mel RIC due to concurrent fungal infection at the time of HCT (n = 5), organ dysfunction (n = 3), prior chemoradiation (n = 2) and Schwachman–Diamond syndrome (n = 1). At the time of allo-HCT, 84% of patients (n = 286) were in first complete remission (CR1), and the majority of patients had de novo AML (n = 210; 61%). Donor median age was 40 years (range: 18–77), and 13% (n = 44) of transplants were from a female donor to a male recipient. MUD was the donor source in 58% (n = 198) of transplants. By HCT disease risk index (DRI), 37% (n = 128) of patients were high-very high risk, and 70% (n = 237) of patients were favorable/intermediate risk by Medical Research Council (MRC) cytogenetic classification. Karnofsky performance status (KPS) of 80–100 was recorded in 94% (n = 321) of patients. HCT-CI score ≥3 was seen in 42% (n = 144) of patients.

**Table 1:** Patient and transplant characteristics.

	2008–2012 (N = 105)	2013–2019 (N = 237)	Total (N = 342)	P-value
Age at HCT, years				0.0001
Median (Range)	60 (32–72)	64 (23–78)	63 (23–78)	
Recipient sex				0.51
Male	50 (47.6%)	122 (51.5%)	172 (50.3%)	
Female	55 (52.4%)	115 (48.5%)	170 (49.7%)	
Female donor to male recipient				0.38
Yes	94 (89.5%)	204 (86.1%)	298 (87.1%)	
No	11 (10.5%)	33 (13.9%)	44 (12.9%)	
Donor age				0.9
Median (range)	43 (19–77)	39 (18–73)	40 (18–77)	
Disease status at HCT				0.18
CR1	73 (69.5%)	173 (73%)	246 (71.9%)	
CR2	9 (8.6%)	27 (11.4%)	36 (10.5%)	
CR3+	0 (0%)	4 (1.7%)	4 (1.2%)	
Active disease	23 (21.9%)	33 (13.9%)	56 (16.4%)	
DRI				0.16
Low-intermediate	61 (58.1%)	153 (64.6%)	214 (62.6%)	
High-very high	44 (41.9%)	84 (35.4%)	128 (37.4%)	
AML type				0.26
Not available	16		16 (4.7%)	
De Novo	62 (69.7%)	148 (62.4%)	210 (61.4%)	
Secondary	19 (21.3%)	72 (30.4%)	91 (26.6%)	
Transformed	8 (9%)	17 (7.2%)	25 (7.3%)	
MRD status				
Not available	105	183	288 (84.2%)	
MRD−	0 (0%)	37 (68.5%)	37 (10.8%)	
MRD+	0 (0%)	17 (31.5%)	17 (5%)	
FLT3ITD				
N/A	98 (93.3%)	200 (84.4%)	298 (87.1%)	
Mutant	7 (6.7%)	37 (15.6%)	44 (12.9%)	
NPM1				
N/A	100 (95.2%)	201 (84.8%)	301 (88%)	
Mutant	5 (4.8%)	36 (15.2%)	41 (12%)	
IDH				
N/A	105 (100%)	209 (88.2%)	314 (91.8%)	
Mutant	0 (0%)	28 (11.8%)	28 (8.2%)	
Karnofsky performance status %				0.002
80–100	105 (100%)	216 (91.1%)	321 (93.9%)	
<80	0 (0%)	21 (8.9%)	21 (6.1%)	
HCT comorbidity index				<0.0001
0	50 (47.6%)	47 (19.8%)	97 (28.4%)	
1–2	31 (29.5%)	70 (29.5%)	101 (29.5%)	
3–4	20 (19%)	86 (36.3%)	106 (31%)	
5+	4 (3.8%)	34 (14.3%)	38 (11.1%)	
Donor type				0.77
MRD	43 (41%)	101 (42.6%)	144 (42.1%)	
MUD	62 (59%)	136 (57.4%)	198 (57.9%)	
ABO blood group compatibility				0.002
ABO compatible	44 (41.9%)	148 (62.4%)	192 (56.1%)	
Minor mismatch (donor is O)	24 (22.9%)	44 (18.6%)	68 (19.9%)	
Major mismatch (recipient is O)	29 (27.6%)	31 (13.1%)	60 (17.5%)	
Bidirectional (none are O)	8 (7.6%)	14 (5.9%)	22 (6.4%)	
Donor/recipient CMV serostatus				0.066
N/A		1	1 (0.3%)	
D−/R−	6 (5.7%)	31 (13.1%)	37 (10.8%)	
D−/R+	40 (38.1%)	74 (31.4%)	114 (33.3%)	
D+/R−	13 (12.4%)	17 (7.2%)	30 (8.8%)	
D+/R+	46 (43.8%)	114 (48.3%)	160 (46.8%)	
Stem cell source				
Peripheral blood stem cells	105 (100%)	237 (100%)	342 (100%)	

*AML* acute myeloid leukemia, *CMV* cytomegalovirus, *CR* complete remission, *DRI* disease risk index, *HCT* hematopoietic cell transplantation, *MRD* minimum residual disease

### Transplant outcomes

**Fig. 1:**
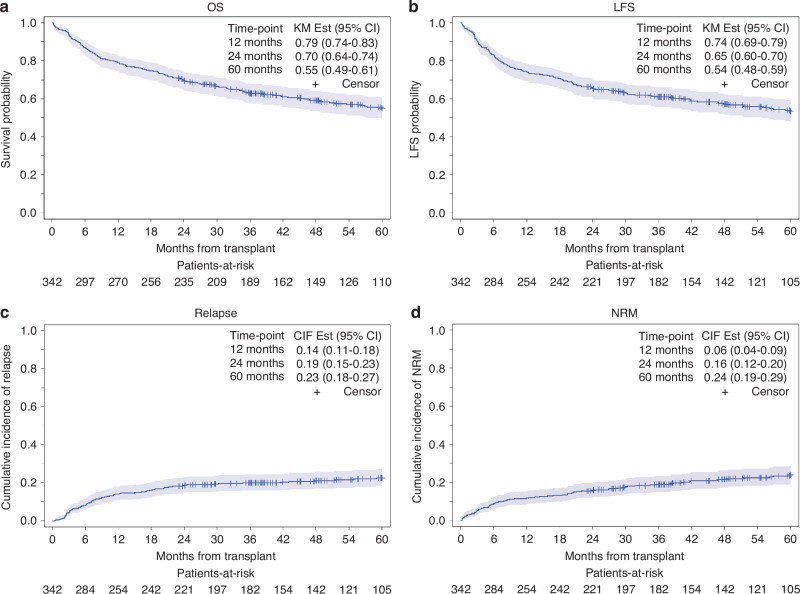
Kaplan Meier curves for transplant outcomes. **a** Overall survival, **b** relapse-free survival, **c** cumulative incidence of relapse, and **d** non-relapse mortality.

With median follow-up of 5.0 years (range: 1.9–13.5 years) in the entire cohort, 5-year OS and LFS were 55% (95% CI: 49–61%) and 54% (95% CI: 48–59%), respectively (Fig. 1a, b), and 5-year CIR and NRM were 23% (95% CI: 18–27%) and 24% (95% CI: 19–29%), respectively (Fig. 1c, d). The median time to neutrophil and platelet engraftment was 15 days (95% CI: 14–15) and 13 (95% CI: 13–14) days, respectively (Supplementary Fig. 1a).

At day +100 post-HCT, cumulative incidence of grade II–IV and grade III–IV aGVHD were 37% (95% CI: 32–42%) and 13% (95% CI: 10–17%), respectively. The 2-year cumulative incidence of cGVHD was 69% (95% CI: 64–74%), and 21% (n = 86) of patients required systemic steroids for management of cGVHD (median dose = 10 mg/day; range: 2–60 mg/day) at 1-year post-HCT. A total of 165 deaths were reported during long-term follow up. The most common causes of death were infection (32%; n = 52), relapse (27%; n = 45), organ failure (10%; n = 17), GVHD-related (4%; n = 7), secondary malignancy (3%; n = 5), and unknown (21%; n = 34).

### Factors influencing transplant outcomes

On UVA, 5-year OS was significantly influenced by active disease vs CR pre*-*HCT [37.9% vs 58.6%, HR = 1.97 (95% CI: 1.37–2.84), p < 0.001], KPS ≤ 70 vs >70 [28% vs 56%, HR = 2.12 (95% CI: 1.21–3.72), p = 0.023], and high-very high DRI vs low-intermediate DRI [50% vs 58%, HR = 1.42 (95% CI: 1.05–1.94; p = 0.023] (Table 2). On MVA, only active disease vs CR pre-HCT retained its influence on 5-year OS [HR = 1.94 (95% CI: 1.33–2.83); p < 0.001] (Table 3).

**Table 2:** Univariate analysis of transplant outcomes.

			**Overall survival**				**LFS**				
		N	**5 years (95% CI)**	**HR (95% CI)**		**P**^**a**^	**5 years (95% CI)**		**HR (95% CI)**		**P**^**a**^
Active disease	CR	286	0.59 (0.52, 0.64)	Reference		**<0.001**	0.57 (0.50, 0.63)		Reference		**<0.001**
	Active	56	0.38 (0.25, 0.51)	1.97 (1.37, 2.84)			0.37 (0.24, 0.50)		1.94 (1.35, 2.80)		
DRI	Low-int	214	0.58 (0.51, 0.65)	Reference		**0.023**	0.56 (0.49, 0.63)		Reference		**0.035**
High-very high		128	0.50 (0.41, 0.59)	1.42 (1.05, 1.94)			0.49 (0.39, 0.57)		1.39 (1.02, 1.88)		
KPS	90–100	224	0.57 (0.49, 0.63)	Reference		**0.022**	0.55 (0.48, 0.62)		Reference		**0.035**
	80	97	0.57 (0.46, 0.66)	0.99 (0.70, 1.41)			0.55 (0.44, 0.64)		1.00 (0.70, 1.41)		
	≤70	21	0.29 (0.10, 0.51)	2.12 (1.21, 3.72)			0.29 (0.10, 0.51)		2.02 (1.15, 3.55)		
			**Relapse**			**NRM**					
		**N**	**5 year (95% CI)**	**HR (95% CI)**	**P**^**b**^	**100 day (95% CI)**		**5 year (95% CI)**		**HR (95% CI)**	**P**^**b**^
Age	≤60	138	0.27 (0.20, 0.35)	Reference	**0.033**	0.03 (0.01, 0.07)		0.20 (0.13, 0.27)		Reference	0.068
(years)	61–69	156	0.23 (0.16, 0.30)	0.77 (0.48, 1.22)		0.07 (0.04, 0.12)		0.28 (0.21, 0.35)		1.56 (1.00, 2.42)	
	≥70	48	0.08 (0.03, 0.19)	0.28 (0.10, 0.79)		0.10 (0.04, 0.21)		0.21 (0.11, 0.34)		1.54 (0.82, 2.87)	
Active disease	CR	286	0.23 (0.18, 0.28)	Reference	0.92	0.04 (0.02, 0.07)		0.21 (0.16, 0.26)		Reference	**<0.001**
	Active	56	0.22 (0.12, 0.34)	0.97 (0.52, 1.81)		0.14 (0.07, 0.25)		0.41 (0.27, 0.54)		2.48 (1.57, 3.92)	
Donor type	MRD	144	0.25 (0.18, 0.33)	Reference	0.35	0.04 (0.01, 0.07)		0.20 (0.14, 0.28)		Reference	**0.03**
	MUD	198	0.21 (0.15, 0.27)	0.80 (0.51, 1.26)		0.08 (0.04, 0.12)		0.27 (0.20, 0.33)		1.56 (1.02, 2.38)	
ABO match	Compatibl	192	0.22 (0.16, 0.28)	Reference	**0.011**	0.07 (0.04, 0.11)		0.24 (0.18, 0.31)		Reference	0.06
	Minor	68	0.13 (0.07, 0.23)	0.60 (0.29, 1.26)		0.06 (0.02, 0.13)		0.37 (0.25, 0.49)		1.68 (1.06, 2.65)	
	Major	60	0.27 (0.16, 0.39)	1.29 (0.73, 2.29)		0.03(0.01, 0.10)		0.12 (0.05, 0.21)		0.85 (0.48, 1.51)	
	Bidir	22	0.49 (0.24, 0.69)	2.54 (1.29, 5.00)		0.05 (0.00, 0.19)		0.14 (0.03, 0.32)		0.67 (0.24, 1.87)	
HCT era	2008–12	105	0.30 (0.21, 0.38)	Reference	**0.025**	0.04 (0.01, 0.09)		0.21 (0.14, 0.29)		Reference	0.5
	2013–19	237	0.19 (0.14, 0.25)	0.58 (0.37, 0.92)		0.07 (0.04, 0.10)		0.25 (0.19, 0.32)		1.00 (0.66, 1.51)	
			**Neutrophil engraftment**				**Platelet engraftment**				
		**N**	**28 day (95% CI)**	**HR (95% CI)**		**P**^**b**^	**28 day (95% CI)**		**HR (95% CI)**		**P**^**b**^
Age	≤60	138	0.97 (0.92, 0.99)	Reference		**0.006**	0.93 (0.87, 0.96)		Reference		0.063
(years)	61–69	156	0.98 (0.94, 0.99)	1.16 (0.94, 1.42)			0.90 (0.84, 0.94)		0.81 (0.66, 1.00)		
	≥70	48	1.00	1.52 (1.20, 1.93)			0.92 (0.78, 0.97)		1.06 (0.76, 1.47)		
Active disease	CR	286	0.99 (0.97, 1.00)	Reference		**0.005**	0.95 (0.92, 0.97)		Reference		**<0.001**
	Active	56	0.93 (0.81, 0.98)	0.66 (0.49, 0.90)			0.71 (0.57, 0.82)		0.47 (0.36, 0.61)		
DRI	Low-int	214	0.99 (0.96, 1.00)	Reference		0.22	0.95 (0.91, 0.97)		Reference		**0.002**
	High-VH	128	0.96 (0.90, 0.98)	0.90 (0.73, 1.10)			0.85 (0.78, 0.90)		0.70 (0.56, 0.86)		
KPS	90–100	224	0.98 (0.94, 0.99)	Reference		0.082	0.95 (0.91, 0.97)		Reference		**<0.001**
	80	97	0.98 (0.89, 1.00)	1.00 (0.81, 1.24)			0.89 (0.80, 0.94)		0.73 (0.58, 0.91)		
	≤70	21	1.00	1.33 (0.86, 2.05)			0.62 (0.37, 0.79)		0.35 (0.24, 0.52)		
			**Grade II–IV aGVHD**				**Grade III–IV aGVHD**				
		**N**	**5 year (95% CI)**	**HR (95% CI)**		**P**^**b**^	**5 year (95% CI)**		**HR (95% CI)**		**P**^**b**^
Active disease	CR	286	0.34 (0.29, 0.40)	Reference		**0.028**	0.11 (0.07, 0.14)		Reference		**0.003**
	Active	56	0.50 (0.36, 0.62)	1.60 (1.06, 2.40)			0.25 (0.15, 0.37)		2.54 (1.36, 4.75)		
Donor type	MRD	144	0.28 (0.21, 0.35)	Reference		**0.003**	0.08 (0.05, 0.14)		Reference		**0.029**
	MUD	198	0.43 (0.36, 0.50)	1.74 (1.21, 2.51)			0.16 (0.11, 0.22)		2.07 (1.08, 3.98)		
Donor age (years)	≤39	169	0.45 (0.37, 0.52)	Reference		**<0.001**	0.16 (0.11, 0.22)		Reference		0.057
	≥40	173	0.29 (0.22, 0.36)	0.55 (0.39, 0.79)			0.10 (0.06, 0.15)		0.57 (0.31, 1.02)		
ABO match	Compatibl	192	0.34 (0.28, 0.41)	Reference		**0.003**	0.13 (0.09, 0.18)		Reference		0.35
	Minor	68	0.52 (0.39, 0.63)	1.66 (1.11, 2.47)			0.15 (0.08, 0.24)		1.09 (0.53,2.22)		
	Major	60	0.22 (0.12, 0.33)	0.59 (0.32, 1.08)			0.07 (0.02, 0.15)		0.48 (0.17,1.37)		
	Bidir	22	0.55 (0.31, 0.73)	1.77 (0.96, 3.27)			0.23 (0.08, 0.42)		1.60 (0.60,4.26)		
CMV status	D−/R−	37	0.46 (0.29, 0.61)	Reference		0.05	0.22 (0.10, 0.36)		Reference		**0.039**
	D−/R+	114	0.44 (0.35, 0.53)	0.95 (0.55, 1.64)			0.18 (0.11, 0.25)		0.81 (0.36, 1.82)		
	D+/R−	30	0.37 (0.20, 0.54)	0.76 (0.37, 1.56)			0.10 (0.03, 0.24)		0.43 (0.11, 1.58)		
	D+/R+	160	0.30 (0.23, 0.37)	0.57 (0.33, 0.99)			0.08 (0.05, 0.13)		0.35 (0.14, 0.83)		
			**Any cGVHD**				**Extensive cGVHD**				
		**N**	**5 year (95% CI)**	**HR (95% CI)**		**P**^**b**^	**5 year (95% CI)**		**HR (95% CI)**		**P**^**b**^
F to M HCT	No	298	0.54 (0.48, 0.59)	Reference		0.24	0.46 (0.40,0.52)		Reference		**0.04**
	Yes	44	0.65 (0.49, 0.78)	1.24 (0.89, 1.72)			0.63 (0.46,0.76)		1.46 (1.04, 2.05)		

Significant p-values were in bold (<0.05).

^a^Based on log-rank tests; only significant factors reported.

^b^Based on Gray’s tests; only significant factors reported.

**Table 3:** Multivariate analysis of transplant outcomes.

			Overall survival		LFS	
		N	Adjusted HR (95% CI)^a^	P^a^	Adjusted HR (95% CI)^a^	P^a^
Active disease	CR	286	Reference	**<0.001**	Reference	**<0.001**
	Active	56	1.94 (1.33, 2.83)		1.95 (1.36, 2.81)	
			**Relapse**		**NRM**	
		**N**	**Adjusted HR (95% CI)**^**b**^	**P**^**b**^	**Adjusted HR (95% CI)**^**b**^	**P**^**b**^
Active disease	CR	286	Reference	0.51	Reference	**<0.001**
	Active	56	0.81 (0.43, 1.52)		2.52 (1.59, 3.99)	
KPS	90–100	224	Reference	**0.042**	Reference	0.43
	80	97	1.36 (0.79, 2.34)		0.73 (0.45, 1.19)	
	≤70	21	2.79 (1.25, 6.25)		0.81 (0.30, 2.15)	
Donor type	MRD	144	Reference	0.84	Reference	**0.049**
	MUD	198	0.95 (0.56, 1.60)		1.56 (1.00, 2.43)	
F to M HCT	No	298	Reference	0.22	Reference	**0.049**
	Yes	44	0.57 (0.23, 1.41)		1.66 (1.00, 2.74)	
ABO match	Compatible	192	Reference	**0.046**	Reference	0.052
	Minor	68	0.55 (0.27, 1.14)		1.49 (0.94, 2.38)	
	Major	60	1.07 (0.61, 1.87)		0.74 (0.43, 1.27)	
	Bidir	22	2.10 (1.02, 4.34)		0.53 (0.18, 1.51)	
HCT era	2008–12	105	Reference	**0.033**	Reference	0.99
	2013–19	237	0.58 (0.35, 0.96)		1.00 (0.65, 1.54)	
			**Neutrophil engraftment**		**Platelet engraftment**	
		**N**	**Adjusted HR (95% CI)**^**c**^	**P**^**c**^	**Adjusted HR (95% CI)**^**c**^	**P**^**c**^
Age	≤60	138	Reference	**0.003**	Reference	0.34
(years)	61–69	156	1.20 (0.97, 1.47)		0.91 (0.74, 1.12)	
	≥70	48	1.53 (1.19, 1.96)		1.14 (0.82, 1.58)	
Active disease	CR	286	Reference	**0.005**	Reference	**<0.001**
	Active	56	0.65 (0.48, 0.88)		0.52 (0.39, 0.69)	
KPS	90–100	224	Reference	0.41	Reference	**<0.001**
	80	97	1.05 (0.85, 1.31)		0.78 (0.62, 0.98)	
	≤70	21	1.34 (0.86, 2.09)		0.40 (0.27, 0.60)	
			**Grade II-IV aGVHD**		**Grade III-IV aGVHD**	
		**N**	**Adjusted HR (95% CI)**^**d**^	**P**^**d**^	**Adjusted HR (95% CI)**^**d**^	**P**^**d**^
Active disease	CR	286	Reference	0.079	Reference	**0.005**
	Active	56	1.43 (0.96, 2.15)		2.41 (1.30, 4.46)	
Donor type	MRD	144	Reference	0.50	Reference	**0.042**
	MUD	198	1.22 (0.69, 2.15)		1.96 (1.02, 3.75)	
Donor age (Years)	≤39	169	Reference	**<0.001**	Reference	0.70
	≥40	173	0.54 (0.38, 0.77)		0.86 (0.41, 1.83)	
ABO match	Compatibl	192	Reference	**0.003**	Reference	0.33
	Minor	68	1.51 (1.01, 2.26)		0.92 (0.45, 1.88)	
	Major	60	0.49 (0.26, 0.93)		0.40 (0.14, 1.16)	
	Bidir	22	1.60 (0.87, 2.93)		1.30 (0.48, 3.53)	
HCT era	2008-12	105	Reference	**0.037**	Reference	0.58
	2013-19	237	0.67 (0.46, 0.98)		0.84(0.45, 1.57)	
			**Any cGVHD**		**Extensive cGVHD**	
		**N**	**Adjusted HR (95% CI)**^**e**^	**P**^**e**^	**Adjusted HR (95% CI)**^**e**^	**P**^**e**^
F to M HCT	No	298	Reference	0.11	Reference	**0.015**
	Yes	44	1.32 (0.94, 1.85)		1.50(1.08, 2.07)	
Cytogenetics	Fav-Int	237	Reference	**0.046**	Reference	0.31
	Adverse	89	0.73 (0.54, 0.99)		0.85 (0.62, 1.16)	

Significant p-values were in bold (<0.05).

^a^Based on multivariable Cox regression model adjusted for active disease. In addition, KPS was adjusted for OS.

^b^Based on multivariable fine and gray regression model. Age, KPS, ABO match, and HCT era were adjusted for relapse. Active disease, donor type, F to M HCT, and ABO were adjusted for NRM.

^c^Based on multivariable fine and gray regression model. Age and active disease were adjusted for neutrophil engraftment. Active disease and KPS were adjusted for platelet engraftment.

^d^Based on multivariable Fine and Gray regression models. Active disease, donor age, ABO and HCT era were adjusted for Grade II-IV aGVHD. Active disease and donor type were adjusted for Grade III-IV aGVHD.

^e^Based on multivariable Fine and Gray regression models. Age and donor type was adjusted for any cGVHD. F to M HCT and ABO match were adjusted for extensive cGVHD.

Similar to OS, on UVA, 5-year LFS was significantly influenced by active disease vs CR pre-HCT [37% vs 56%, HR = 1.94 (95% CI: 1.35–2.80); p < 0.001], KPS ≤ 70 vs >70 [28% vs 55%, HR = 2.02 (95% CI: 1.15–3.55); p = 0.035] and high-very high vs low-intermediate DRI [48% vs 56%, HR = 1.39 (95% CI: 1.02–1.88); p < 0.035] (Table 2), but only active disease vs CR pre-HCT [HR = 1.95 (95% CI: 1.36–2.81); p < 0.001] retained its influence on 5-year LFS on MVA (Table 3).

On UVA, 5-year NRM was significantly influenced by active disease vs CR pre-HCT [41% vs 21%; HR = 2.48; (95% CI: 1.57–3.92); p = 0.001] and MUD vs MRD [26% vs 20%; HR = 1.56 (95% CI: 1.02–2.38); p = 0.030]. However, on MVA, only female-to-male HCT [HR = 1.66 (95% CI: 1.00–2.74); p = 0.049], active disease vs CR [HR = 2.52 (95% CI: 1.59–3.99); p = 0.001], and MUD vs MRD [HR = 1.56 (95% CI: 1.00–2.43); p = 0.049] significantly influenced 5-year NRM (Table 3).

### Subgroup analysis

In patients ≥70 years old (n = 48, median age: 71, range 70–78), 5-year OS, LFS, and NRM were 70% (95% CI: 55–81%), 70% (95% CI: 55–81%), and 21% (95% CI: 11–34%), respectively, which was comparable to younger patients in this study (p = 0.29, 0.19 and 0.48, respectively) and confirms our earlier reports on Mel-based RIC in patients ≥70 years old [22]. CIR at 5 years was significantly lower in older patients (8%, 95%CI: 3-18%) compared to younger patients (25%, 95% CI: 20–30%; p = 0.019). Patients who were older also had significantly more unrelated donors (77% vs 55%; p = 0.004) and AML from antecedent prior hematologic disease (AHD: 39% vs 26%; p = 0.027) compared to younger HCT recipients. The 5-year OS, LFS, and NRM in patients who were in CR1 at the time of allo-HCT (n = 246) were 59% (95% CI: 52–65%), 57% (95% CI: 50–63%), and 22% (95% CI: 17–28%), respectively. This was significantly better than those in CR2+ (n = 96), with 5-year OS, LFS, and NRM of 46% (95% CI: 35–56%; p = 0.005), 46% (95% CI: 35–56%; p = 0.008), and 28% (95% CI:19–38%; p = 0.033), respectively. MRD status by multicolor flow cytometry (performed at University of Washington, Brentwood) was available for 54 patients who were in morphologic remission and transplanted after 2018. Patients with MRD+ status pre-HCT (n = 17) had significantly lower 5-year OS [36% vs 62%, HR = 2.75 (95% CI: 1.18–6.36); p = 0.014] and LFS [41% vs 62%; HR = 2.67 (95% CI: 1.15–6.21), p = 0.016] and higher 5-year NRM [29% vs 22%; HR = 2.25 (95%CI: 0.70–7.24); p = 0.18] compared to those with MRD- status. Targeted PCR sequencing data for select somatic mutations were available for a subgroup of patients: FLT3-ITD (n = 50), NPM1 (n = 47), and IDH1 or 2 (n = 30). The 5-year OS in patients with FLT3-ITD, NPM1 and IDH mutations was 67%, 59%, and 83%, respectively.

### Secular trends

Recent era HCT recipients (n = 237, 69%) were significantly older (64 vs 60 years; p = 0.0001), had higher HCT-CI ≥ 3 (42% vs 23%; p < 0.0001), lower KPS ≤ 80 (9% vs none; p = 0.002), and less major ABO incompatibility (13% vs 27%; p = 0.002) compared to recipients in the early era (Table 1). There was no significant difference in DRI scores (37% vs 42%; p = 0.16), donor median age (39 vs 43 years; p = 0.9), or utilization of unrelated donors (59% vs 57%; p = 0.77). Similarly, no differences were noted in the number of patients with secondary AML (30% vs 21%; p = 0.26) or in CR1 (73% vs 70%; p = 0.16) at the time of allo-HCT. OS at 5 years was similar (53% vs 56%, p = 0.88), despite adverse demographic factors in the recent era.

## Discussion

With the development of new safe and effective induction regimens for AML, more older patients are achieving CR and are eligible for HCT [23]. As such, it is important to identify a RIC regimen and GVHD prophylaxis strategy that can optimally balance disease control and NRM, resulting in favorable long-term outcomes for older patients with AML. In prior CIBMTR analyses of AML patients, Flu/Mel conditioning was associated with inferior OS in the first 3 months due to higher NRM and aGVHD rates compared to Flu/Bu [3]. More studies have reported increased early NRM in patients with AML/MDS who receive Flu/Mel [24–26] with CNI/MTX. At City of Hope, we have successfully combined Flu/Mel with Tac/Sir GVHD prophylaxis and reported acceptably low NRM in MDS, myelofibrosis, and acute lymphoblastic leukemia [16, 18].

Results of our single-institution retrospective analysis of patients with AML who underwent allo-HCT with Flu/Mel and Tac/Sir demonstrated promising 5-year OS/LFS of 55% and 54%, respectively. This compares favorably with the outcomes reported by the CIBMTR in AML patients undergoing allo-HCT with either RIC [3] or MAC [4]. This regimen is also associated with low NRM at day +100 and 2 years (6% and 16%, respectively).

In patients ≥70 years old (n = 48), we report 5-year OS, LFS, and NRM of 70%, 70%, and 21%, respectively, showing good safety and efficacy of Flu/Mel+ Tac/Siro regimen in this subgroup, and confirming our earlier report [22]. These results may be explained by (i) Favorable cytogenetics/mutations in majority of patients. MRC favorable/good risk cytogenetics was seen in 80% (n = 38) of older patients. After risk stratification by including somatic mutations, 35% (n = 17) of patients were upstaged to ELN 2022 adverse risk, while 64% of patients (n = 31) remained classified as ELN 2022 intermediate risk. (ii) Few ≥70 year old patients had active disease pre-HCT (4%, n = 2) or high/very high DRI scores (8%, n = 4), (iii) Better pre-HCT disease control based on MRD status which was available for 37% (n = 18) of these patients, and majority of patients 66% (n = 12) were MRD- pre-HCT. (iv) Dose of Mel: 75% (n = 36) of ≥70 year old patients received Mel 140 mg/m^2^, which may have contributed to leukemia eradication (v) Resilience of older AML patients: The majority of ≥70 year old 69% (n = 33) received intensive induction chemotherapy at AML diagnosis, 90% (n = 42) had HCT-CI < 5 and 94% (n = 45) of patients KPS > 80%, respectively. These factors could explain favorable 5-year NRM of 21% seen in our study.

On MVA, active disease at the time of HCT was predictive of inferior 5-year OS/LFS. Despite emerging evidence [27], our data suggest that disease control prior to HCT is still a major factor impacting OS, especially in patients receiving RIC [28, 29]. Thus, outcomes for patients with AML who have residual disease pre-HCT may be improved through intensification of conditioning with total body irradiation (helical tomotherapy) [30] or maintenance chemotherapy [31] together with comprehensive geriatric assessments [32].

Cumulative incidence of cGVHD at 1 year was relatively high at 55%, resulting in a low 1-year GRFS rate of 37%. However, 2-year NRM was only 16%, and only 21% patients were on prednisone at 1-year post-HCT [median dose of 10 mg/day; range: 2–60 mg/day]. This may reflect improvements in clinical outcomes due to novel strategies for management of cGVHD. Alternatively, presence of cGVHD and use of Sir may be protective against AML relapse due to the enhanced graft-versus-leukemia effect conferred by cGVHD [33] and inhibition of the mTOR pathway by Sir [34], especially in high-risk patients [35]. Efforts are ongoing to reduce the incidence of GVHD, and we and others have conducted trials to improve outcomes with Tac/Sir prophylaxis by addition of anti-thymocyte globulin [36], JAK inhibitors [37] and IL12/IL23 blocking antibody, ustekinumab [38]. More recently, encouraging data has emerged from results of a randomized study (BMT-CTN 1703) showing superior 1-year GRFS (52% vs 35%; HR = 0.64; p = 0.001) in patients who received post-transplant cyclophosphamide compared to Tac/MTX-based GVHD prophylaxis [39]. However, follow-up from this study is short, and patients who received post-transplant cyclophosphamide had slower engraftment and more infection [40]. T cell receptor deep sequencing data also showed lower T cell receptor diversity at day +28 post-HCT that may persist for up to 2 years after transplant [41]. Hence, follow-up results beyond 1 year are awaited to see if benefit is maintained [42].

Finally, patients transplanted in the recent era, when compared to early era had similar 5-year OS, suggesting that risk factors for relapse/NRM (older age, high HCT-CI, lower KPS) may be overcome with Flu/Mel plus Tac/Sir and advances in supportive care practices [43, 44].

We acknowledge several limitations for our study, including its single-center design and lack of data on pretransplant MRD status for all patients. This is primarily due to lack of reliable MRD testing in the early part of our study. Another limitation of our study is its retrospective design, which includes patients transplanted over an extended time period without available molecular data, ultimately limiting the novelty of the findings. Despite these limitations, our data show that Flu/Mel with Tac/Sir prophylaxis results in favorable long-term OS and acceptable NRM in patients with AML, especially those ≥70 years old. Our study also identified areas of future development, allowing this regimen to serve as a platform in studies attempting to lower GVHD rates seen in our study.

## Supplementary information

Supplementary Figure 1

Supplementary Figure 2

Supplementary Table 1

Supplementary Table 2

Supplementary Table 3

## Data Availability

For original data please contact asalhotra@coh.org. Individual participant data will not be shared.
