# Supplementary figures and images for: Long-term survival outcomes of allo-HCT in AML with fludarabine/melphalan conditioning and tacrolimus/sirolimus GVHD prophylaxis
Source: Bone Marrow Transplant. 2025 Nov 18;61(1):82–91. doi: 10.1038/s41409-025-02738-4 (PMC12819140; doi:10.1038/s41409-025-02738-4)

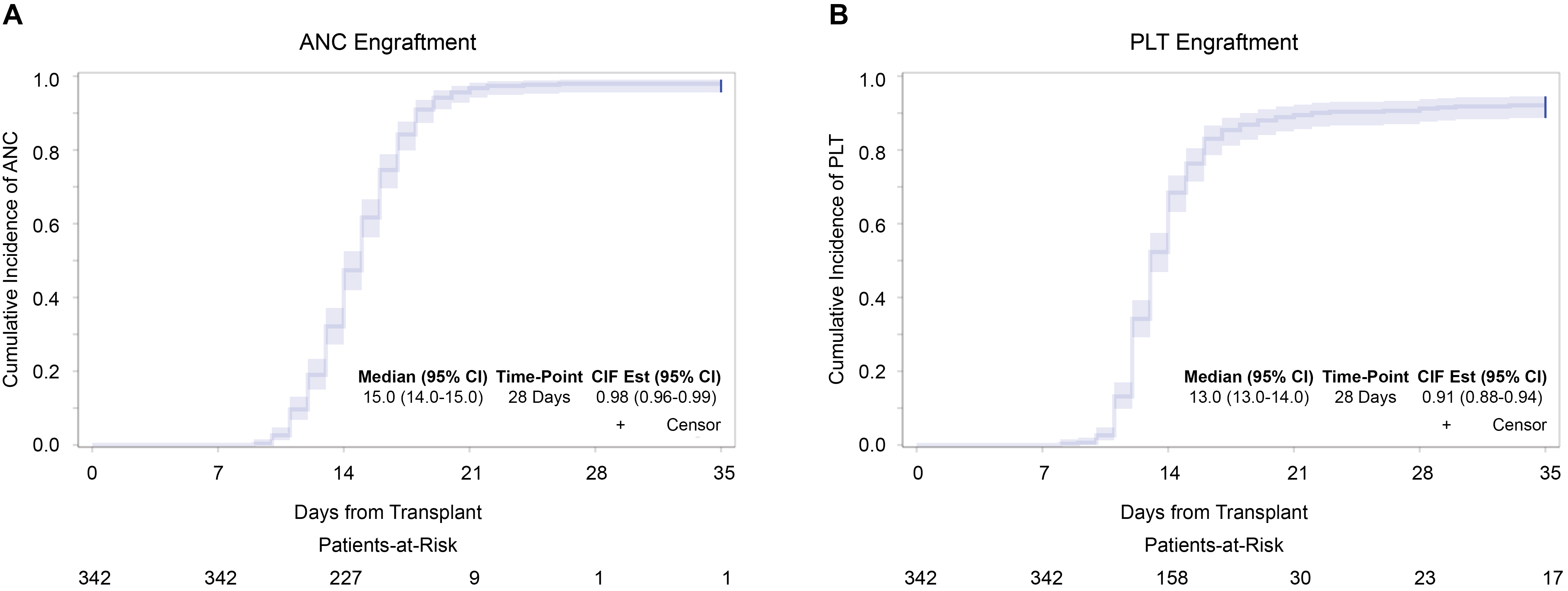

Supplement: Supplementary file 1 — Supplementary Figure 1 [file 41409_2025_2738_MOESM1_ESM.png]

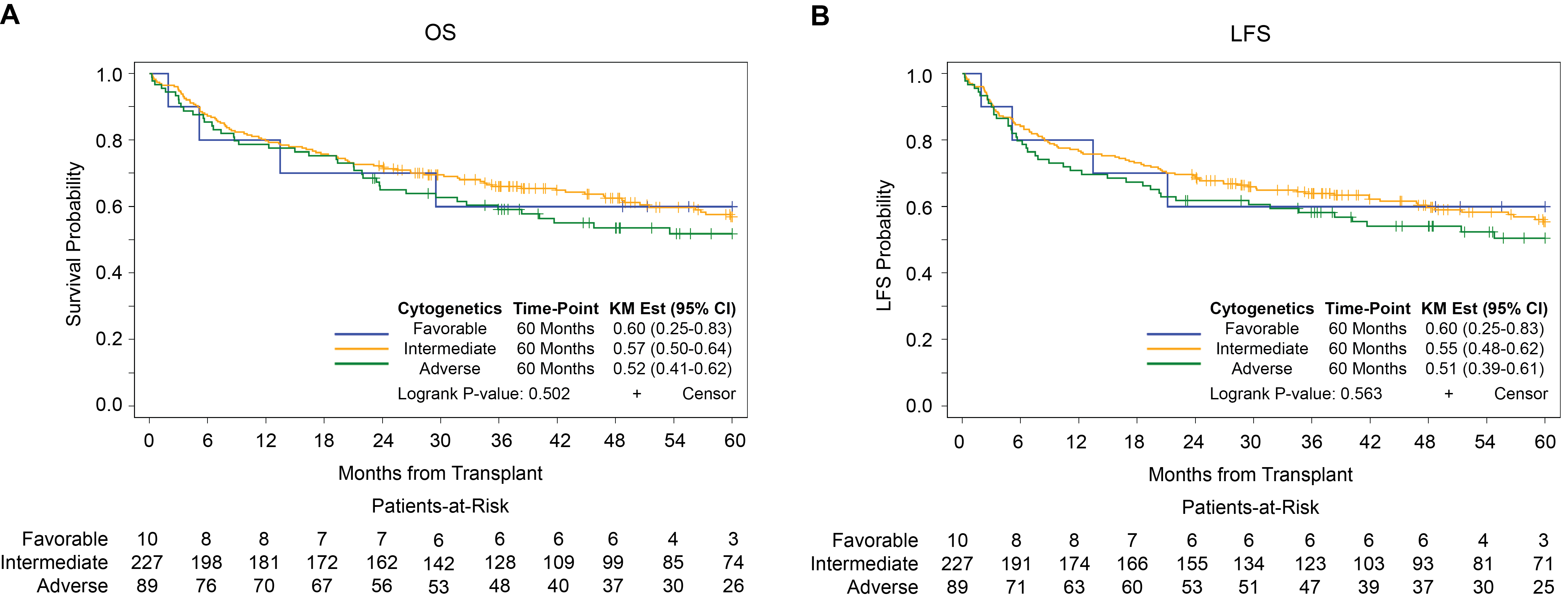

Supplement: Supplementary file 2 — Supplementary Figure 2 [file 41409_2025_2738_MOESM2_ESM.png]
