# Supplementary material for: Long-term survival outcomes of allo-HCT in AML with fludarabine/melphalan conditioning and tacrolimus/sirolimus GVHD prophylaxis
Source: Bone Marrow Transplant. 2025 Nov 18;61(1):82–91. doi: 10.1038/s41409-025-02738-4 (PMC12819140; doi:10.1038/s41409-025-02738-4)
Supplement: Supplementary file 4 — Supplementary Table 2 [file 41409_2025_2738_MOESM4_ESM.docx]

|  | | | *Overall Survival* | | | *RFS* | | |
| --- | --- | --- | --- | --- | --- | --- | --- | --- |
|  |  | *N* | *5 Yr (95%CI)* | *HR (95%CI)* | *P** | *5 Yr (95%CI)* | *HR (95%CI)* | *P** |
| Age, years | ≤60 | 138 | 0.548(0.456,0.630) | Reference | 0.36 | 0.527(0.436,0.610) | Reference | 0.24 |
|  | 61-69 | 156 | 0.516(0.429,0.596) | 1.17(0.84,1.62) |  | 0.498(0.412,0.578) | 1.18(0.86,1.63) |  |
|  | ≥70 | 48 | 0.701(0.547,0.811) | 0.83(0.49,1.41) |  | 0.703(0.550,0.813) | 0.78(0.46,1.32) |  |
| Sex | M | 172 | 0.578(0.493,0.654) | Reference | 0.56 | 0.557(0.472,0.635) | Reference | 0.68 |
|  | F | 170 | 0.522(0.440,0.597) | 1.09(0.81,1.49) |  | 0.510(0.430,0.584) | 1.07(0.79,1.44) |  |
| Active Disease | CR | 286 | 0.586(0.522,0.644) | Reference | **<0.001** | 0.567(0.503,0.626) | Reference | **<0.001** |
|  | Active | 56 | 0.379(0.246,0.510) | 1.97(1.37,2.84) |  | 0.371(0.242,0.501) | 1.94(1.35,2.80) |  |
| Cytogenetics | Fav-Int | 237 | 0.570(0.499,0.635) | Reference | 0.24 | 0.556(0.485,0.621) | Reference | 0.29 |
|  | Adverse | 89 | 0.519(0.405,0.620) | 1.23(0.87,1.73) |  | 0.505(0.391,0.608) | 1.20(0.85,1.69) |  |
| DRI | Low-int | 214 | 0.581(0.507,0.649) | Reference | **0.023** | 0.563(0.489,0.632) | Reference | **0.035** |
|  | High-VH | 128 | 0.500(0.406,0.587) | 1.42(1.05,1.94) |  | 0.487(0.394,0.574) | 1.39(1.02,1.88) |  |
| KPS | 90-100 | 224 | 0.567(0.494,0.633) | Reference | **0.022** | 0.552(0.480,0.619) | Reference | **0.035** |
|  | 80 | 97 | 0.568(0.458,0.664) | 0.99(0.70,1.41) |  | 0.547(0.437,0.643) | 1.00(0.70,1.41) |  |
|  | ≤70 | 21 | 0.286(0.096,0.512) | 2.12(1.21,3.72) |  | 0.286(0.096,0.512) | 2.02(1.15,3.55) |  |
| HCTCI | 0 | 97 | 0.593(0.484,0.686) | Reference | 0.88 | 0.579(0.472,0.671) | Reference | 0.89 |
|  | 1-2 | 101 | 0.528(0.420,0.625) | 1.00(0.67,1.49) |  | 0.516(0.407,0.614) | 0.95(0.64,1.41) |  |
|  | 3-4 | 106 | 0.537(0.428,0.634) | 1.07(0.72,1.59) |  | 0.517(0.408,0.616) | 1.04(0.70,1.53) |  |
|  | ≥5 | 38 | 0.538(0.352,0.692) | 1.22(0.71,2.11) |  | 0.503(0.317,0.662) | 1.17(0.69,1.99) |  |
| Donor type | MRD | 144 | 0.573(0.482,0.655) | Reference | 0.14 | 0.545(0.454,0.628) | Reference | 0.28 |
|  | MUD | 198 | 0.536(0.460,0.606) | 1.26(0.92,1.73) |  | 0.528(0.452,0.598) | 1.19(0.87,1.62) |  |
| Donor Age, yrs | ≤39 | 169 | 0.562(0.478,0.638) | Reference | 0.93 | 0.554(0.470,0.630) | Reference | 0.69 |
|  | ≥40 | 173 | 0.540(0.458,0.615) | 1.01(0.75,1.38) |  | 0.517(0.435,0.593) | 1.06(0.78,1.44) |  |
| F to M HCT | No | 298 | 0.539(0.477,0.597) | Reference | 0.77 | 0.520(0.458,0.579) | Reference | 0.68 |
|  | Yes | 44 | 0.636(0.467,0.764) | 0.93(0.59,1.48) |  | 0.637(0.469,0.764) | 0.91(0.57,1.43) |  |
| ABO match | Compatibl | 192 | 0.555(0.476,0.628) | Reference | 0.49 | 0.543(0.464,0.615) | Reference | 0.42 |
|  | Minor | 68 | 0.510(0.381,0.625) | 1.13(0.76,1.66) |  | 0.494(0.366,0.610) | 1.13(0.77,1.66) |  |
|  | Major | 60 | 0.655(0.516,0.763) | 1.02(0.67,1.55) |  | 0.617(0.475,0.731) | 1.01(0.67,1.54) |  |
|  | Bidir | 22 | 0.370(0.163,0.580) | 1.53(0.87,2.70) |  | 0.379(0.171,0.587) | 1.59(0.90,2.79) |  |
| CMV serostatus | D-/R- | 37 | 0.521(0.343,0.672) | Reference | 0.80 | 0.525(0.347,0.675) | Reference | 0.81 |
|  | D-/R+ | 114 | 0.565(0.464,0.654) | 1.01(0.59,1.74) |  | 0.539(0.439,0.630) | 1.06(0.62,1.82) |  |
|  | D+/R- | 30 | 0.475(0.268,0.657) | 1.11(0.56,2.19) |  | 0.475(0.268,0.657) | 1.12(0.57,2.22) |  |
|  | D+/R+ | 160 | 0.568(0.482,0.645) | 0.88(0.52,1.50) |  | 0.551(0.465,0.628) | 0.92(0.54,1.56) |  |
| HCT era | 2008-12 | 105 | 0.533(0.434,0.623) | Reference | 0.37 | 0.495(0.397,0.586) | Reference | 0.17 |
|  | 2013-19 | 237 | 0.567(0.496,0.632) | 0.88(0.63,1.22) |  | 0.555(0.482,0.621) | 0.82(0.59,1.13) |  |

* Based on log-rank test.

|  | | | *Relapse* | | | *NRM* | | | |
| --- | --- | --- | --- | --- | --- | --- | --- | --- | --- |
|  |  | *N* | *5 Yr (95%CI)* | *HR (95%CI)* | *P** | *100 day (95%CI)* | *5 Yr (95%CI)* | *HR (95%CI)* | *P** |
| Age, years | ≤60 | 138 | 0.274(0.201,0.352) | Reference | **0.033** | 0.029(0.010,0.068) | 0.199(0.133,0.274) | Reference | 0.068 |
|  | 61-69 | 156 | 0.225(0.159,0.297) | 0.77(0.48,1.22) |  | 0.071(0.037,0.118) | 0.277(0.206,0.352) | 1.56(1.00,2.42) |  |
|  | ≥70 | 48 | 0.084(0.026,0.185) | 0.28(0.10,0.79) |  | 0.104(0.038,0.210) | 0.213(0.109,0.340) | 1.54(0.82,2.87) |  |
| Sex | M | 172 | 0.192(0.134,0.259) | Reference | 0.15 | 0.058(0.030,0.100) | 0.250(0.182,0.324) | Reference | 0.26 |
|  | F | 170 | 0.259(0.194,0.328) | 1.40(0.89,2.21) |  | 0.059(0.030,0.101) | 0.231(0.169,0.299) | 0.80(0.53,1.20) |  |
| Active Disease | CR | 286 | 0.226(0.178,0.278) | Reference | 0.92 | 0.042(0.023,0.070) | 0.207(0.159,0.259) | Reference | **<0.001** |
|  | Active | 56 | 0.221(0.120,0.342) | 0.97(0.52,1.81) |  | 0.143(0.066,0.248) | 0.407(0.273,0.538) | 2.48(1.57,3.92) |  |
| Cytogenetics | Fav-Int | 237 | 0.190(0.141,0.245) | Reference | 0.089 | 0.051(0.028,0.084) | 0.254(0.196,0.315) | Reference | 0.62 |
|  | Adverse | 89 | 0.284(0.190,0.385) | 1.55(0.94,2.54) |  | 0.079(0.034,0.147) | 0.211(0.131,0.304) | 0.90(0.56,1.45) |  |
| DRI | Low-int | 214 | 0.215(0.161,0.275) | Reference | 0.62 | 0.037(0.018,0.069) | 0.222(0.164,0.284) | Reference | 0.074 |
|  | High-VH | 128 | 0.243(0.170,0.323) | 1.13(0.71,1.79) |  | 0.094(0.051,0.152) | 0.270(0.193,0.352) | 1.46(0.97,2.19) |  |
| KPS | 90-100 | 224 | 0.208(0.155,0.267) | Reference | 0.14 | 0.045(0.023,0.078) | 0.240(0.183,0.301) | Reference | 0.60 |
|  | 80 | 97 | 0.230(0.151,0.319) | 1.18(0.70,1.96) |  | 0.062(0.025,0.122) | 0.223(0.142,0.317) | 0.83(0.52,1.33) |  |
|  | ≤70 | 21 | 0.381(0.176,0.585) | 2.16(1.02,4.61) |  | 0.190(0.057,0.383) | 0.333(0.112,0.577) | 1.16(0.48,2.79) |  |
| HCTCI | 0 | 97 | 0.217(0.141,0.305) | Reference | 0.76 | 0.031(0.008,0.081) | 0.204(0.128,0.292) | Reference | 0.28 |
|  | 1-2 | 101 | 0.259(0.175,0.352) | 1.09(0.61,1.95) |  | 0.069(0.030,0.130) | 0.225(0.144,0.317) | 0.79(0.45,1.37) |  |
|  | 3-4 | 106 | 0.201(0.127,0.288) | 0.80(0.43,1.46) |  | 0.057(0.023,0.112) | 0.282(0.192,0.378) | 1.21(0.74,1.98) |  |
|  | ≥5 | 38 | 0.250(0.111,0.418) | 0.89(0.41,1.95) |  | 0.105(0.033,0.227) | 0.247(0.119,0.399) | 1.32(0.66,2.64) |  |
| Donor type | MRD | 144 | 0.254(0.184,0.331) | Reference | 0.35 | 0.035(0.013,0.074) | 0.200(0.135,0.275) | Reference | **0.030** |
|  | MUD | 198 | 0.205(0.150,0.267) | 0.80(0.51,1.26) |  | 0.076(0.044,0.118) | 0.266(0.204,0.332) | 1.56(1.02,2.38) |  |
|  |  | 169 | 0.200(0.141,0.267) | Reference | 0.25 | 0.065(0.034,0.109) | 0.246(0.181,0.316) | Reference | 0.47 |
|  |  | 173 | 0.251(0.187,0.319) | 1.31(0.83,2.06) |  | 0.052(0.026,0.092) | 0.232(0.169,0.302) | 0.88(0.59,1.32) |  |
| F to M HCT | No | 298 | 0.239(0.190,0.291) | Reference | 0.18 | 0.064(0.040,0.095) | 0.241(0.191,0.294) | Reference | 0.32 |
|  | Yes | 44 | 0.136(0.055,0.255) | 0.57(0.25,1.35) |  | 0.023(0.002,0.105) | 0.227(0.110,0.369) | 1.32(0.79,2.21) |  |
| ABO match | Compatibl | 192 | 0.218(0.160,0.282) | Reference | **0.011** | 0.068(0.038,0.109) | 0.239(0.177,0.306) | Reference | 0.060 |
|  | Minor | 68 | 0.132(0.065,0.225) | 0.60(0.29,1.26) |  | 0.059(0.019,0.133) | 0.373(0.254,0.493) | 1.68(1.06,2.65) |  |
|  | Major | 60 | 0.267(0.156,0.390) | 1.29(0.73,2.29) |  | 0.033(0.006,0.103) | 0.117(0.051,0.213) | 0.85(0.48,1.51) |  |
|  | Bidir | 22 | 0.485(0.240,0.693) | 2.54(1.29,5.00) |  | 0.045(0.003,0.194) | 0.136(0.032,0.316) | 0.67(0.24,1.87) |  |
| CMV serostatus | D-/R- | 37 | 0.189(0.082,0.330) | Reference | 0.21 | 0.081(0.020,0.198) | 0.286(0.144,0.446) | Reference | 0.26 |
|  | D-/R+ | 114 | 0.200(0.130,0.281) | 1.01(0.43,2.37) |  | 0.053(0.021,0.105) | 0.261(0.180,0.348) | 1.15(0.56,2.36) |  |
|  | D+/R- | 30 | 0.353(0.177,0.534) | 2.10(0.82,5.41) |  | 0.067(0.011,0.195) | 0.172(0.049,0.359) | 0.52(0.18,1.55) |  |
|  | D+/R+ | 160 | 0.223(0.159,0.293) | 1.10(0.49,2.49) |  | 0.056(0.028,0.100) | 0.227(0.162,0.298) | 0.85(0.42,1.75) |  |
| HCT era | 2008-12 | 105 | 0.295(0.211,0.384) | Reference | **0.025** | 0.038(0.012,0.088) | 0.210(0.137,0.292) | Reference | 0.50 |
|  | 2013-19 | 237 | 0.193(0.142,0.249) | 0.58(0.37,0.92) |  | 0.068(0.040,0.104) | 0.252(0.194,0.315) | 1.00(0.66,1.51) |  |

* Based on Gray’s test.

|  | | | *Neutrophil Engraftment* | | | *Platelet Engraftment* | | |
| --- | --- | --- | --- | --- | --- | --- | --- | --- |
|  |  | *N* | *28 Day (95%CI)* | *HR (95%CI)* | *P** | *28 Day (95%CI)* | *HR (95%CI)* | *P** |
| Age, years | ≤60 | 138 | 0.971(0.915,0.990) | Reference | **0.006** | 0.928(0.868,0.961) | Reference | 0.063 |
|  | 61-69 | 156 | 0.981(0.937,0.994) | 1.16(0.94,1.42) |  | 0.897(0.837,0.936) | 0.81(0.66,1.00) |  |
|  | ≥70 | 48 | 1.000 | 1.52(1.20,1.93) |  | 0.917(0.781,0.970) | 1.06(0.76,1.47) |  |
| Sex | M | 172 | 0.971(0.929,0.988) | Reference | 0.79 | 0.901(0.845,0.938) | Reference | 0.24 |
|  | F | 170 | 0.988(0.937,0.998) | 1.02(0.85,1.23) |  | 0.924(0.871,0.955) | 1.12(0.93,1.36) |  |
| Active Disease | CR | 286 | 0.990(0.968,0.997) | Reference | **0.005** | 0.951(0.918,0.971) | Reference | **<0.001** |
|  | Active | 56 | 0.929(0.806,0.975) | 0.66(0.49,0.90) |  | 0.714(0.574,0.816) | 0.47(0.36,0.61) |  |
| Cytogenetics | Fav-Int | 237 | 0.983(0.956,0.994) | Reference | 0.57 | 0.916(0.872,0.945) | Reference | 0.63 |
|  | Adverse | 89 | 0.966(0.887,0.990) | 0.98(0.77,1.23) |  | 0.921(0.838,0.963) | 0.94(0.76,1.18) |  |
| DRI | Low-int | 214 | 0.991(0.963,0.998) | Reference | 0.22 | 0.949(0.908,0.972) | Reference | **0.002** |
|  | High-VH | 128 | 0.961(0.903,0.984) | 0.90(0.73,1.10) |  | 0.852(0.776,0.903) | 0.70(0.56,0.86) |  |
| KPS | 90-100 | 224 | 0.978(0.944,0.991) | Reference | 0.082 | 0.951(0.912,0.973) | Reference | **<0.001** |
|  | 80 | 97 | 0.979(0.891,0.996) | 1.00(0.81,1.24) |  | 0.887(0.802,0.936) | 0.73(0.58,0.91) |  |
|  | ≤70 | 21 | 1.000 | 1.33(0.86,2.05) |  | 0.619(0.371,0.793) | 0.35(0.24,0.52) |  |
| HCTCI | 0 | 97 | 0.979(0.909,0.995) | Reference | 0.77 | 0.948(0.875,0.979) | Reference | 0.30 |
|  | 1-2 | 101 | 0.980(0.895,0.996) | 1.02(0.80,1.29) |  | 0.901(0.822,0.946) | 0.88(0.69,1.13) |  |
|  | 3-4 | 106 | 0.991(0.858,0.999) | 1.11(0.88,1.40) |  | 0.906(0.829,0.949) | 0.81(0.65,1.02) |  |
|  | ≥5 | 38 | 0.947(0.737,0.990) | 0.95(0.66,1.37) |  | 0.868(0.700,0.946) | 0.77(0.53,1.13) |  |
| Donor type | MRD | 144 | 1.000 | Reference | 0.76 | 0.938(0.881,0.968) | Reference | 0.29 |
|  | MUD | 198 | 0.965(0.925,0.984) | 0.97(0.81,1.16) |  | 0.894(0.841,0.930) | 0.90(0.75,1.09) |  |
| Donor Age, yrs | ≤39 | 169 | 0.970(0.929,0.988) | Reference | 0.55 | 0.899(0.842,0.937) | Reference | 0.45 |
|  | ≥40 | 173 | 0.988(0.938,0.998) | 1.05(0.87,1.27) |  | 0.925(0.873,0.956) | 1.08(0.89,1.30) |  |
| F to M HCT | No | 298 | 0.980(0.953,0.991) | Reference | 0.58 | 0.909(0.870,0.937) | Reference | 0.98 |
|  | Yes | 44 | 0.977(0.756,0.998) | 1.10(0.83,1.44) |  | 0.932(0.785,0.980) | 1.00(0.81,1.24) |  |
| ABO match | Compatibl | 192 | 0.990(0.944,0.998) | Reference | 0.070 | 0.927(0.879,0.957) | Reference | 0.25 |
|  | Minor | 68 | 0.985(0.785,0.999) | 1.24(0.96,1.60) |  | 0.912(0.808,0.961) | 0.96(0.75,1.23) |  |
|  | Major | 60 | 0.933(0.826,0.975) | 0.84(0.62,1.13) |  | 0.867(0.746,0.932) | 0.74(0.58,0.95) |  |
|  | Bidir | 22 | 1.000(1.000,1.000) | 1.02(0.73,1.44) |  | 0.909(0.627,0.981) | 0.78(0.56,1.10) |  |
| CMV serostatus | D-/R- | 37 | 1.000 | Reference | 0.96 | 0.946(0.765,0.989) | Reference | 0.27 |
|  | D-/R+ | 114 | 0.974(0.920,0.991) | 0.94(0.71,1.24) |  | 0.895(0.820,0.939) | 0.77(0.58,1.02) |  |
|  | D+/R- | 30 | 0.967(0.566,0.998) | 0.88(0.58,1.35) |  | 0.900(0.694,0.970) | 0.80(0.54,1.17) |  |
|  | D+/R+ | 160 | 0.981(0.936,0.995) | 0.98(0.75,1.28) |  | 0.919(0.863,0.952) | 0.93(0.71,1.22) |  |
| HCT era | 2008-12 | 105 | 0.962(0.894,0.987) | Reference | 0.89 | 0.924(0.851,0.962) | Reference | 0.80 |
|  | 2013-19 | 237 | 0.987(0.954,0.997) | 1.01(0.81,1.26) |  | 0.907(0.862,0.938) | 0.97(0.80,1.19) |  |

* Based on Gray’s test.

|  | | | *Grade II-IV aGVHD* | | | *Grade III-IV aGVHD* | | |
| --- | --- | --- | --- | --- | --- | --- | --- | --- |
|  |  | *N* | *100 days (95%CI)* | *HR (95%CI)* | *P** | *100 days (95%CI)* | *HR (95%CI)* | *P** |
| Age, years | ≤60 | 138 | 0.362(0.283,0.442) | Reference | 0.30 | 0.109(0.064,0.167) | Reference | 0.56 |
|  | 61-69 | 156 | 0.404(0.326,0.480) | 1.16(0.80,1.67) |  | 0.141(0.092,0.201) | 1.33(0.70,2.55) |  |
|  | ≥70 | 48 | 0.271(0.154,0.402) | 0.75(0.42,1.35) |  | 0.146(0.063,0.261) | 1.49(0.65,3.41) |  |
| Sex | M | 172 | 0.360(0.289,0.432) | Reference | 0.56 | 0.157(0.107,0.215) | Reference | 0.17 |
|  | F | 170 | 0.376(0.304,0.449) | 1.10(0.78,1.55) |  | 0.100(0.061,0.151) | 0.66(0.37,1.19) |  |
| Active Disease | CR | 286 | 0.343(0.288,0.398) | Reference | **0.028** | 0.105(0.073,0.144) | Reference | **0.003** |
|  | Active | 56 | 0.500(0.362,0.623) | 1.60(1.06,2.40) |  | 0.250(0.145,0.369) | 2.54(1.36,4.75) |  |
| Cytogenetics | Fav-Int | 237 | 0.350(0.290,0.411) | Reference | 0.27 | 0.118(0.081,0.163) | Reference | 0.35 |
|  | Adverse | 89 | 0.427(0.322,0.527) | 1.23(0.85,1.79) |  | 0.157(0.090,0.241) | 1.34(0.71,2.53) |  |
| DRI | Low-int | 214 | 0.327(0.265,0.390) | Reference | 0.056 | 0.103(0.067,0.148) | Reference | 0.067 |
|  | High-VH | 128 | 0.438(0.350,0.522) | 1.39(0.99,1.97) |  | 0.172(0.112,0.242) | 1.70(0.95,3.04) |  |
| KPS | 90-100 | 224 | 0.362(0.299,0.424) | Reference | 0.59 | 0.129(0.089,0.177) | Reference | 0.97 |
|  | 80 | 97 | 0.402(0.304,0.498) | 1.11(0.76,1.61) |  | 0.124(0.067,0.198) | 0.94(0.48,1.83) |  |
|  | ≤70 | 21 | 0.286(0.112,0.488) | 0.72(0.32,1.61) |  | 0.143(0.034,0.327) | 1.07(0.33,3.45) |  |
| HCTCI | 0 | 97 | 0.371(0.275,0.467) | Reference | 0.76 | 0.134(0.075,0.210) | Reference | 0.44 |
|  | 1-2 | 101 | 0.376(0.282,0.470) | 1.02(0.65,1.59) |  | 0.168(0.103,0.248) | 1.27(0.62,2.60) |  |
|  | 3-4 | 106 | 0.387(0.294,0.479) | 1.05(0.67,1.64) |  | 0.094(0.048,0.159) | 0.68(0.30,1.55) |  |
|  | ≥5 | 38 | 0.289(0.155,0.439) | 0.74(0.38,1.44) |  | 0.105(0.033,0.227) | 0.77(0.25,2.35) |  |
| Donor type | MRD | 144 | 0.278(0.207,0.353) | Reference | **0.003** | 0.083(0.045,0.136) | Reference | **0.029** |
|  | MUD | 198 | 0.434(0.364,0.502) | 1.74(1.21,2.51) |  | 0.162(0.114,0.216) | 2.07(1.08,3.98) |  |
| Donor Age, yrs | ≤39 | 169 | 0.450(0.373,0.523) | Reference | **<0.001** | 0.160(0.109,0.219) | Reference | 0.057 |
|  | ≥40 | 173 | 0.289(0.223,0.358) | 0.55(0.39,0.79) |  | 0.098(0.060,0.148) | 0.57(0.31,1.02) |  |
| F to M HCT | No | 298 | 0.376(0.321,0.431) | Reference | 0.28 | 0.128(0.093,0.168) | Reference | 0.94 |
|  | Yes | 44 | 0.318(0.186,0.458) | 0.76(0.45,1.28) |  | 0.136(0.055,0.255) | 1.00(0.43,2.33) |  |
| ABO match | Compatibl | 192 | 0.344(0.277,0.411) | Reference | **0.003** | 0.130(0.087,0.182) | Reference | 0.35 |
|  | Minor | 68 | 0.515(0.389,0.627) | 1.66(1.11,2.47) |  | 0.147(0.075,0.242) | 1.09(0.53,2.22) |  |
|  | Major | 60 | 0.217(0.122,0.328) | 0.59(0.32,1.08) |  | 0.067(0.021,0.149) | 0.48(0.17,1.37) |  |
|  | Bidir | 22 | 0.545(0.312,0.729) | 1.77(0.96,3.27) |  | 0.227(0.080,0.419) | 1.60(0.60,4.26) |  |
| CMV serostatus | D-/R- | 37 | 0.459(0.292,0.611) | Reference | 0.050 | 0.216(0.100,0.361) | Reference | **0.039** |
|  | D-/R+ | 114 | 0.439(0.346,0.527) | 0.95(0.55,1.64) |  | 0.175(0.112,0.251) | 0.81(0.36,1.82) |  |
|  | D+/R- | 30 | 0.367(0.198,0.537) | 0.76(0.37,1.56) |  | 0.100(0.025,0.239) | 0.43(0.11,1.58) |  |
|  | D+/R+ | 160 | 0.300(0.231,0.372) | 0.57(0.33,0.99) |  | 0.081(0.045,0.130) | 0.35(0.14,0.83) |  |
| HCT era | 2008-12 | 105 | 0.429(0.332,0.521) | Reference | 0.080 | 0.152(0.091,0.228) | Reference | 0.36 |
|  | 2013-19 | 237 | 0.342(0.282,0.402) | 0.73(0.51,1.05) |  | 0.118(0.081,0.163) | 0.76(0.41,1.40) |  |

* Based on Gray’s test.

|  | | | *Any cGVHD* | | | *Extensive cGVHD* | | |
| --- | --- | --- | --- | --- | --- | --- | --- | --- |
|  |  | *N* | *1-Yr (95%CI)* | *HR (95%CI)* | *P** | *1 Yr (95%CI)* | *HR (95%CI)* | *P** |
| Age, years | ≤60 | 138 | 0.580(0.492,0.657) | Reference | 0.29 | 0.529(0.442,0.609) | Reference | 0.14 |
|  | 61-69 | 156 | 0.532(0.450,0.607) | 0.84(0.64,1.10) |  | 0.449(0.369,0.525) | 0.78(0.58,1.03) |  |
|  | ≥70 | 48 | 0.521(0.369,0.653) | 0.77(0.54,1.11) |  | 0.437(0.293,0.573) | 0.74(0.50,1.09) |  |
| Sex | M | 172 | 0.593(0.515,0.663) | Reference | 0.097 | 0.506(0.428,0.578) | Reference | 0.42 |
|  | F | 170 | 0.506(0.428,0.578) | 0.82(0.64,1.05) |  | 0.453(0.377,0.526) | 0.90(0.69,1.17) |  |
| Active Disease | CR | 286 | 0.542(0.482,0.598) | Reference | 0.90 | 0.462(0.403,0.518) | Reference | 0.39 |
|  | Active | 56 | 0.589(0.446,0.707) | 1.04(0.70,1.55) |  | 0.571(0.429,0.691) | 1.20(0.80,1.80) |  |
| Cytogenetics | Fav-Int | 237 | 0.570(0.504,0.630) | Reference | 0.077 | 0.494(0.428,0.556) | Reference | 0.23 |
|  | Adverse | 89 | 0.517(0.407,0.616) | 0.76(0.56,1.03) |  | 0.472(0.364,0.572) | 0.83(0.60,1.13) |  |
| DRI | Low-int | 214 | 0.551(0.482,0.615) | Reference | 0.37 | 0.463(0.394,0.528) | Reference | 0.95 |
|  | High-VH | 128 | 0.547(0.456,0.629) | 0.89(0.68,1.16) |  | 0.508(0.418,0.591) | 0.99(0.75,1.32) |  |
| KPS | 90-100 | 224 | 0.567(0.499,0.629) | Reference | 0.21 | 0.482(0.415,0.546) | Reference | 0.33 |
|  | 80 | 97 | 0.515(0.411,0.610) | 0.83(0.63,1.09) |  | 0.485(0.381,0.580) | 0.94(0.70,1.25) |  |
|  | ≤70 | 21 | 0.524(0.282,0.719) | 0.62(0.31,1.24) |  | 0.429(0.209,0.632) | 0.59(0.27,1.28) |  |
| HCTCI | 0 | 97 | 0.536(0.431,0.630) | Reference | 0.50 | 0.464(0.362,0.560) | Reference | 0.15 |
|  | 1-2 | 101 | 0.505(0.403,0.598) | 1.06(0.76,1.48) |  | 0.396(0.300,0.490) | 0.95(0.67,1.36) |  |
|  | 3-4 | 106 | 0.557(0.456,0.646) | 1.12(0.81,1.54) |  | 0.519(0.419,0.610) | 1.22(0.87,1.71) |  |
|  | ≥5 | 38 | 0.684(0.505,0.810) | 1.37(0.86,2.19) |  | 0.632(0.453,0.766) | 1.48(0.91,2.40) |  |
| Donor type | MRD | 144 | 0.549(0.463,0.626) | Reference | 0.19 | 0.486(0.402,0.565) | Reference | 0.33 |
|  | MUD | 198 | 0.551(0.478,0.617) | 1.18(0.92,1.52) |  | 0.475(0.403,0.543) | 1.15(0.88,1.50) |  |
| Donor Age, yrs | ≤39 | 169 | 0.562(0.484,0.633) | Reference | 0.11 | 0.479(0.402,0.552) | Reference | 0.44 |
|  | ≥40 | 173 | 0.538(0.460,0.609) | 0.82(0.64,1.05) |  | 0.480(0.403,0.552) | 0.90(0.69,1.17) |  |
| F to M HCT | No | 298 | 0.537(0.478,0.592) | Reference | 0.24 | 0.460(0.402,0.515) | Reference | **0.041** |
|  | Yes | 44 | 0.636(0.472,0.762) | 1.23(0.89,1.70) |  | 0.614(0.450,0.742) | 1.45(1.04,2.02) |  |
| ABO match | Compatibl | 192 | 0.542(0.468,0.609) | Reference | 0.28 | 0.458(0.386,0.527) | Reference | 0.10 |
|  | Minor | 68 | 0.676(0.549,0.775) | 1.33(0.96,1.84) |  | 0.632(0.504,0.736) | 1.47(1.04,2.07) |  |
|  | Major | 60 | 0.467(0.335,0.588) | 1.13(0.82,1.57) |  | 0.417(0.290,0.538) | 1.25(0.89,1.75) |  |
|  | Bidir | 22 | 0.455(0.236,0.651) | 0.91(0.54,1.52) |  | 0.364(0.168,0.564) | 0.91(0.52,1.58) |  |
| CMV serostatus | D-/R- | 37 | 0.378(0.223,0.533) | Reference | 0.30 | 0.378(0.223,0.533) | Reference | 0.51 |
|  | D-/R+ | 114 | 0.579(0.482,0.664) | 1.56(0.98,2.47) |  | 0.509(0.413,0.597) | 1.28(0.81,2.03) |  |
|  | D+/R- | 30 | 0.567(0.365,0.726) | 1.54(0.88,2.70) |  | 0.400(0.223,0.571) | 0.95(0.51,1.75) |  |
|  | D+/R+ | 160 | 0.569(0.488,0.642) | 1.54(0.99,2.42) |  | 0.500(0.420,0.575) | 1.27(0.81,1.99) |  |
| HCT era | 2008-12 | 105 | 0.543(0.442,0.633) | Reference | 0.89 | 0.457(0.359,0.550) | Reference | 0.75 |
|  | 2013-19 | 237 | 0.553(0.487,0.614) | 0.97(0.74,1.27) |  | 0.489(0.424,0.551) | 1.03(0.77,1.38) |  |

* Based on Gray’s test.
