# Supplementary material for: Long-term survival outcomes of allo-HCT in AML with fludarabine/melphalan conditioning and tacrolimus/sirolimus GVHD prophylaxis
Source: Bone Marrow Transplant. 2025 Nov 18;61(1):82–91. doi: 10.1038/s41409-025-02738-4 (PMC12819140; doi:10.1038/s41409-025-02738-4)
Supplement: Supplementary file 5 — Supplementary Table 3 [file 41409_2025_2738_MOESM5_ESM.docx]

|  | | | *Overall Survival* | | *RFS* | | *Relapse* | | *NRM* | |
| --- | --- | --- | --- | --- | --- | --- | --- | --- | --- | --- |
|  |  | *N* | *Adjusted HR (95%CI)** | *P** | *Adjusted HR (95%CI)** | *P** | *Adjusted HR (95%CI)* † | *P*† | *Adjusted HR (95%CI)* † | *P*† |
| Age, years | ≤60 | 138 | Reference | 0.57 | Reference | 0.36 | Reference | 0.089 | Reference | 0.58 |
|  | 61-69 | 156 | 1.06(0.75,1.48) |  | 1.08(0.78,1.50) |  | 0.74(0.46,1.19) |  | 1.27(0.79,2.03) |  |
|  | ≥70 | 48 | 0.79(0.47,1.36) |  | 0.74(0.43,1.26) |  | 0.34(0.12,0.96) |  | 1.26(0.65,2.43) |  |
| Sex | M | 172 | Reference | 0.46 | Reference | 0.57 | Reference | 0.29 | Reference | 0.66 |
|  | F | 170 | 1.12(0.83,1.52) |  | 1.09(0.81,1.48) |  | 1.29(0.81,2.05) |  | 0.90(0.57,1.43) |  |
| Active Disease | CR | 286 | Reference | **<0.001** | Reference | **<0.001** | Reference | 0.51 | Reference | **<0.001** |
|  | Active | 56 | 1.94(1.33,2.83) |  | 1.95(1.36,2.81) |  | 0.81(0.43,1.52) |  | 2.52(1.59,3.99) |  |
| Cytogenetics | Fav-Int | 237 | Reference | 0.47 | Reference | 0.29 | Reference | 0.56 | Reference | 0.59 |
|  | Adverse | 89 | 1.14(0.80,1.63) |  | 1.20(0.85,1.70) |  | 1.18(0.67,2.09) |  | 0.88(0.54,1.41) |  |
| DRI | Low-int | 214 | Reference | 0.87 | Reference | 0.84 | Reference | 0.71 | Reference | 0.53 |
|  | High-VH | 128 | 1.03(0.69,1.53) |  | 1.04(0.70,1.54) |  | 0.91(0.55,1.49) |  | 0.84(0.49,1.44) |  |
| KPS | 90-100 | 224 | Reference | 0.072 | Reference | 0.10 | Reference | **0.042** | Reference | 0.43 |
|  | 80 | 97 | 0.89(0.62,1.27) |  | 0.90(0.63,1.28) |  | 1.36(0.79,2.34) |  | 0.73(0.45,1.19) |  |
|  | ≤70 | 21 | 1.80(1.01,3.18) |  | 1.72(0.97,3.04) |  | 2.79(1.25,6.25) |  | 0.81(0.30,2.15) |  |
| HCTCI | 0 | 97 | Reference | 0.97 | Reference | 0.98 | Reference | 0.87 | Reference | 0.73 |
|  | 1-2 | 101 | 1.01(0.68,1.52) |  | 0.97(0.65,1.44) |  | 1.04(0.57,1.90) |  | 0.84(0.48,1.46) |  |
|  | 3-4 | 106 | 1.03(0.69,1.53) |  | 1.02(0.69,1.51) |  | 0.82(0.44,1.54) |  | 1.12(0.68,1.85) |  |
|  | ≥5 | 38 | 1.15(0.66,2.01) |  | 1.08(0.64,1.85) |  | 0.89(0.42,1.88) |  | 1.24(0.60,2.55) |  |
| Donor type | MRD | 144 | Reference | 0.30 | Reference | 0.36 | Reference | 0.84 | Reference | **0.049** |
|  | MUD | 198 | 1.18(0.86,1.63) |  | 1.16(0.85,1.58) |  | 0.95(0.56,1.60) |  | 1.56(1.00,2.43) |  |
| Donor Age, yrs | ≤39 | 169 | Reference | 0.55 | Reference | 0.50 | Reference | 0.73 | Reference | 0.086 |
|  | ≥40 | 173 | 1.10(0.81,1.51) |  | 1.11(0.82,1.50) |  | 1.09(0.67,1.78) |  | 1.63(0.93,2.85) |  |
| F to M HCT | No | 298 | Reference | 0.88 | Reference | 0.79 | Reference | 0.22 | Reference | **0.049** |
|  | Yes | 44 | 0.97(0.61,1.53) |  | 0.94(0.59,1.48) |  | 0.57(0.23,1.41) |  | 1.66(1.00,2.74) |  |
| ABO match | Compatibl | 192 | Reference | 0.74 | Reference | 0.57 | Reference | **0.046** | Reference | 0.052 |
|  | Minor | 68 | 1.07(0.72,1.58) |  | 1.07(0.73,1.58) |  | 0.55(0.27,1.14) |  | 1.49(0.94,2.38) |  |
|  | Major | 60 | 0.97(0.64,1.47) |  | 0.98(0.64,1.48) |  | 1.07(0.61,1.87) |  | 0.74(0.43,1.27) |  |
|  | Bidir | 22 | 1.35(0.76,2.39) |  | 1.48(0.84,2.61) |  | 2.10(1.02,4.34) |  | 0.53(0.18,1.51) |  |
| CMV serostatus | D-/R- | 37 | Reference | 0.87 | Reference | 0.88 | Reference | 0.22 | Reference | 0.34 |
|  | D-/R+ | 114 | 0.90(0.52,1.55) |  | 0.99(0.57,1.69) |  | 1.00(0.42,2.37) |  | 0.91(0.45,1.83) |  |
|  | D+/R- | 30 | 0.97(0.49,1.94) |  | 0.96(0.48,1.92) |  | 2.03(0.79,5.22) |  | 0.41(0.14,1.19) |  |
|  | D+/R+ | 160 | 0.83(0.49,1.41) |  | 0.87(0.51,1.47) |  | 1.11(0.48,2.57) |  | 0.80(0.39,1.64) |  |
| HCT era | 2008-12 | 105 | Reference | 0.35 | Reference | 0.24 | Reference | **0.033** | Reference | 0.99 |
|  | 2013-19 | 237 | 0.85(0.60,1.20) |  | 0.82(0.60,1.14) |  | 0.58(0.35,0.96) |  | 1.00(0.65,1.54) |  |

* Based on multivariable Cox regression model adjusted for active disease. In addition, KPS was adjusted for OS.

† Based on multivariable Fine and Gray regression model. Age, KPS, ABO match, and HCT era were adjusted for relapse. Active disease, donor type, F to M HCT, and ABO were adjusted for NRM.

|  | | | *Neutrophil Engraftment* | | *Platelet Engraftment* | |
| --- | --- | --- | --- | --- | --- | --- |
|  |  | *N* | *Adjusted HR (95%CI)* † | *P*† | *Adjusted HR (95%CI)* † | *P*† |
| Age, years | ≤60 | 138 | Reference | **0.003** | Reference | 0.34 |
|  | 61-69 | 156 | 1.20(0.97,1.47) |  | 0.91(0.74,1.12) |  |
|  | ≥70 | 48 | 1.53(1.19,1.96) |  | 1.14(0.82,1.58) |  |
| Sex | M | 172 | Reference | 0.54 | Reference | 0.15 |
|  | F | 170 | 1.06(0.88,1.28) |  | 1.16(0.95,1.41) |  |
| Active Disease | CR | 286 | Reference | **0.005** | Reference | **<0.001** |
|  | Active | 56 | 0.65(0.48,0.88) |  | 0.52(0.39,0.69) |  |
| Cytogenetics | Fav-Int | 237 | Reference | 0.64 | Reference | 0.38 |
|  | Adverse | 89 | 1.05(0.84,1.32) |  | 1.11(0.88,1.39) |  |
| DRI | Low-int | 214 | Reference | 0.18 | Reference | 0.37 |
|  | High-VH | 128 | 1.17(0.93,1.48) |  | 1.12(0.88,1.43) |  |
| KPS | 90-100 | 224 | Reference | 0.41 | Reference | **<0.001** |
|  | 80 | 97 | 1.05(0.85,1.31) |  | 0.78(0.62,0.98) |  |
|  | ≤70 | 21 | 1.34(0.86,2.09) |  | 0.40(0.27,0.60) |  |
| HCTCI | 0 | 97 | Reference | 0.79 | Reference | 0.45 |
|  | 1-2 | 101 | 1.03(0.81,1.29) |  | 0.91(0.71,1.15) |  |
|  | 3-4 | 106 | 1.11(0.89,1.40) |  | 0.84(0.67,1.06) |  |
|  | ≥5 | 38 | 0.98(0.67,1.42) |  | 0.81(0.54,1.21) |  |
| Donor type | MRD | 144 | Reference | 0.46 | Reference | 0.42 |
|  | MUD | 198 | 0.93(0.77,1.12) |  | 0.92(0.75,1.12) |  |
| Donor Age, yrs | ≤39 | 169 | Reference | 0.13 | Reference | 0.41 |
|  | ≥40 | 173 | 1.16(0.96,1.41) |  | 1.09(0.89,1.32) |  |
| F to M HCT | No | 298 | Reference | 0.59 | Reference | 0.96 |
|  | Yes | 44 | 1.07(0.83,1.39) |  | 0.99(0.82,1.21) |  |
| ABO match | Compatibl | 192 | Reference | 0.12 | Reference | 0.15 |
|  | Minor | 68 | 1.33(1.03,1.71) |  | 0.98(0.75,1.28) |  |
|  | Major | 60 | 0.90(0.67,1.20) |  | 0.75(0.58,0.97) |  |
|  | Bidir | 22 | 1.08(0.75,1.54) |  | 0.90(0.67,1.19) |  |
| CMV serostatus | D-/R- | 37 | Reference | 0.93 | Reference | 0.38 |
|  | D-/R+ | 114 | 0.97(0.73,1.28) |  | 0.84(0.63,1.13) |  |
|  | D+/R- | 30 | 0.97(0.62,1.51) |  | 0.83(0.54,1.27) |  |
|  | D+/R+ | 160 | 1.03(0.79,1.35) |  | 0.99(0.75,1.32) |  |
| HCT era | 2008-12 | 105 | Reference | 0.29 | Reference | 0.70 |
|  | 2013-19 | 237 | 0.88(0.70,1.11) |  | 1.04(0.84,1.31) |  |

† Based on multivariable Fine and Gray regression model. Age, and active disease were adjusted for neutrophil engraftment. Active disease and KPS were adjusted for platelet engraftment.

|  | | | *Grade II-IV aGVHD* | | *Grade III-IV aGVHD* | | *Any cGVHD* | | *Extensive cGVHD* | |
| --- | --- | --- | --- | --- | --- | --- | --- | --- | --- | --- |
|  |  | *N* | *Adjusted HR (95%CI)** | *P** | *Adjusted HR (95%CI)** | *P** | *Adjusted HR (95%CI)* † | *P*† | *Adjusted HR (95%CI)* † | *P*† |
| Age, years | ≤60 | 138 | Reference | 0.22 | Reference | 0.94 | Reference | 0.12 | Reference | 0.18 |
|  | 61-69 | 156 | 0.97(0.67,1.40) |  | 1.09(0.55,2.14) |  | 0.79(0.60,1.04) |  | 0.78(0.59,1.04) |  |
|  | ≥70 | 48 | 0.58(0.31,1.10) |  | 1.16(0.50,2.71) |  | 0.71(0.49,1.03) |  | 0.76(0.51,1.13) |  |
| Sex | M | 172 | Reference | 0.56 | Reference | 0.21 | Reference | 0.071 | Reference | 0.89 |
|  | F | 170 | 1.11(0.79,1.56) |  | 0.68(0.38,1.23) |  | 0.79(0.61,1.02) |  | 0.98(0.73,1.31) |  |
| Active Disease | CR | 286 | Reference | 0.079 | Reference | **0.005** | Reference | 0.91 | Reference | 0.34 |
|  | Active | 56 | 1.43(0.96,2.15) |  | 2.41(1.30,4.46) |  | 1.02(0.68,1.55) |  | 1.22(0.81,1.85) |  |
| Cytogenetics | Fav-Int | 237 | Reference | 0.52 | Reference | 0.49 | Reference | **0.046** | Reference | 0.31 |
|  | Adverse | 89 | 1.13(0.77,1.67) |  | 1.24(0.67,2.31) |  | 0.73(0.54,0.99) |  | 0.85(0.62,1.16) |  |
| DRI | Low-int | 214 | Reference | 0.53 | Reference | 0.90 | Reference | 0.27 | Reference | 0.87 |
|  | High-VH | 128 | 1.15(0.74,1.80) |  | 1.05(0.47,2.32) |  | 0.86(0.65,1.13) |  | 1.02(0.77,1.35) |  |
| KPS | 90-100 | 224 | Reference | 0.30 | Reference | 0.86 | Reference | 0.42 | Reference | 0.46 |
|  | 80 | 97 | 1.22(0.83,1.79) |  | 0.85(0.43,1.67) |  | 0.87(0.65,1.16) |  | 0.92(0.68,1.23) |  |
|  | ≤70 | 21 | 0.69(0.32,1.50) |  | 0.82(0.28,2.44) |  | 0.68(0.34,1.38) |  | 0.63(0.29,1.37) |  |
| HCTCI | 0 | 97 | Reference | 0.59 | Reference | 0.32 | Reference | 0.61 | Reference | 0.17 |
|  | 1-2 | 101 | 1.13(0.71,1.81) |  | 1.24(0.61,2.52) |  | 1.03(0.74,1.44) |  | 0.98(0.69,1.41) |  |
|  | 3-4 | 106 | 1.17(0.72,1.91) |  | 0.61(0.26,1.42) |  | 1.10(0.80,1.52) |  | 1.27(0.91,1.78) |  |
|  | ≥5 | 38 | 0.74(0.34,1.60) |  | 0.66(0.20,2.15) |  | 1.37(0.84,2.22) |  | 1.52(0.93,2.48) |  |
| Donor type | MRD | 144 | Reference | 0.50 | Reference | **0.042** | Reference | 0.071 | Reference | 0.23 |
|  | MUD | 198 | 1.22(0.69,2.15) |  | 1.96(1.02,3.75) |  | 1.27(0.98,1.65) |  | 1.18(0.90,1.56) |  |
| Donor Age, yrs | ≤39 | 169 | Reference | **<0.001** | Reference | 0.70 | Reference | 0.27 | Reference | 0.24 |
|  | ≥40 | 173 | 0.54(0.38,0.77) |  | 0.86(0.41,1.83) |  | 0.79(0.53,1.19) |  | 0.85(0.65,1.11) |  |
| F to M HCT | No | 298 | Reference | 0.70 | Reference | 0.59 | Reference | 0.11 | Reference | **0.015** |
|  | Yes | 44 | 0.90(0.53,1.53) |  | 1.26(0.55,2.89) |  | 1.32(0.94,1.85) |  | 1.50(1.08,2.07) |  |
| ABO match | Compatibl | 192 | Reference | **0.003** | Reference | 0.33 | Reference | 0.35 | Reference | 0.089 |
|  | Minor | 68 | 1.51(1.01,2.26) |  | 0.92(0.45,1.88) |  | 1.31(0.95,1.82) |  | 1.47(1.04,2.08) |  |
|  | Major | 60 | 0.49(0.26,0.93) |  | 0.40(0.14,1.16) |  | 1.04(0.74,1.47) |  | 1.27(0.91,1.77) |  |
|  | Bidir | 22 | 1.60(0.87,2.93) |  | 1.30(0.48,3.53) |  | 0.89(0.54,1.49) |  | 0.87(0.50,1.53) |  |
| CMV serostatus | D-/R- | 37 | Reference | 0.19 | Reference | 0.072 | Reference | 0.24 | Reference | 0.38 |
|  | D-/R+ | 114 | 0.75(0.42,1.34) |  | 0.65(0.29,1.46) |  | 1.50(0.94,2.38) |  | 1.25(0.77,2.02) |  |
|  | D+/R- | 30 | 0.59(0.28,1.27) |  | 0.36(0.10,1.27) |  | 1.48(0.84,2.61) |  | 0.86(0.46,1.61) |  |
|  | D+/R+ | 160 | 0.56(0.31,0.99) |  | 0.34(0.14,0.82) |  | 1.59(1.02,2.49) |  | 1.26(0.79,2.00) |  |
| HCT era | 2008-12 | 105 | Reference | **0.037** | Reference | 0.58 | Reference | 0.74 | Reference | 0.59 |
|  | 2013-19 | 237 | 0.67(0.46,0.98) |  | 0.84(0.45,1.57) |  | 1.05(0.79,1.40) |  | 1.09(0.81,1.46) |  |

* Based on multivariable Fine and Gray regression models. Active disease, donor age, ABO and HCT era were adjusted for Grade II-IV aGVHD. Active disease and donor type were adjusted for Grade III-IV aGVHD.

† Based on multivariable Fine and Gray regression models. Age and donor type was adjusted for any cGVHD. F to M HCT and ABO match were adjusted for extensive cGVHD.
